# Editorial: Personal Genomes: Accessing, Sharing, and Interpretation

**DOI:** 10.3389/fgene.2021.687584

**Published:** 2021-06-04

**Authors:** Manuel Corpas, Stephan Beck, Gustavo Glusman, Mahsa Shabani

**Affiliations:** ^1^Cambridge Precision Medicine Limited, ideaSpace, University of Cambridge Biomedical Innovation Hub, Cambridge, United Kingdom; ^2^Department of Madingley Hall, Institute of Continuing Education, University of Cambridge, Cambridge, United Kingdom; ^3^Facultad de Ciencias de la Salud, Universidad Internacional de La Rioja, Madrid, Spain; ^4^Department of Cancer Biology, University College London (UCL) Cancer Institute, University College London, London, United Kingdom; ^5^Institute for Systems Biology (ISB), Seattle, WA, United States; ^6^Metamedica, Faculty of Law and Criminology, Ghent University, Ghent, Belgium

**Keywords:** personal genomics, direct-to-consumer genetic testing, citizen science, data sharing, ethical, legal, social implications

Over the past few years we have witnessed a number of advances in the personal genomics space including (a) more affordable sequencing technology, (b) mainstreaming of genomics in healthcare systems, (c) augmented sharing of genomic data, and (d) increased demand for direct-to-consumer genetic testing. All of these developments have brought us closer to the long-awaited genomics revolution. This genomics revolution is not exempt from challenges, in part amplified by lack of standards (ethical, legal, and technological), slow translation of knowledge to the clinic, and unequal access of personal genome benefits for all.

As the vast majority of reference data in public databases continue to be of European ancestries, existing health disparities between rich and poor are likely to continue. In parallel, access to direct-to-consumer personal genetic testing continues to increase the public's appetite for genotyping and ancestry testing, resulting in greater number of enquiries with clinicians and public healthcare systems. This has left many medical professionals unprepared and unable to harness the wave of patient-focused healthcare and demand for data sharing—data sharing which is crucial for establishing better, more precise tools for diagnosis and treatment. However, sharing also opens the door for privacy concerns as secure access of genome data and metadata cannot always be guaranteed. Increased legal protection and institutional support are likely to keep promoting positive impact for diverse participation.

On April 11–12, 2019, we helped organize the First Personal Genomes Conference at the Wellcome Genome Campus, Hinxton, UK, to discuss issues around personal genomic data access, sharing, and interpretation (Rubin and Glusman, 2019). In this Research Topic in *Frontiers in Genetics*, we collected a selection of representative research related to the Conference's topics revolving around the themes of (a) personal genetic testing, (b) interpretation of personal genomes, (c) personal genomics citizen science, (d) return of data to genome research participants, (e) data protection, privacy and the ethics of data sharing, and (f) clinical perspective—from patients to the public.

In our Research Topic, Du and Wang describe how the direct-to-consumer market in China has been particularly buoyant in recent years, with many providers offering multiple channels for purchasing genetic testing products. They argue, however, that a regulatory vacuum exists in how to obtain valid informed consent, and protect customers' genetic data from access by third parties. In India, Pemmasani et al. stress the need for existing data reference repositories to expand their variant data information, offering non-European personal genomes equitable access to resources and tools for their interpretation. Folkersen et al. present *Impute.me*, an open source tool for analyzing direct-to-consumer genetic data. With tools such as *Impute.me* anyone in the world can calculate and interpret polygenic risk scores free of charge. Guerra-Assunção et al. present another tool, *GenomeChronicler*, that uses open access Personal Genome Project (PGP-UK Consortium, 2018) data to generate reports (for research use only) that include information relating to possibly beneficial and harmful variants as well as ancestry. Mehandziska et al. show an analytical pipeline to effectively report variants of unknown significance, which to date remain among the most challenging types of variants to interpret. Corpas et al. illustrate how existing tools and resources can be leveraged for whole genome analysis when combined. Their extensive battery of analyses for a single family provides a use case for clinicians on how to develop healthcare plans for the individual, based on genetic and other healthcare data.

Access to raw personal genomic data in clinical settings is becoming commonplace in many European nations. Narayanasamy et al. explored personal genome access policies and practices from a pool of European sequencing institutions engaged in generating massive amounts of sequencing data. They report a generalized lack of clear policies and processes for raw genomic data retention and access among large sequencing facilities. Wallace et al. argue that even when raw data are available, enabling genomic and biomedical data to be accessed and shared for secondary research purposes is not always straightforward for existing “legacy” datasets. A filter used by researchers could help determine the extent to which a given dataset can be shared. Ahmed and Shabani suggest that data sharing promoted by DNA marketplaces raises concerns about consent and privacy, and may have implications for public-funded research that does not offer incentives to share. Yet, for parents of children suffering from rare and common diseases, there are powerful incentives to share whole genome sequencing data. Beauvais et al. provide recommendations for healthcare professionals in the clinical and research contexts when faced with sharing genomic data on parental request for a child's raw genomic data. Wöhlke et al. contribute to this debate by suggesting that lay people's sharing perceptions are important because they affect both their interest in undertaking genetic testing as well as their interpretations of test results. Their survey on personal sharing preferences in both Germany and Italy shows a relatively high willingness among participants to share information with their social circle, but an overall strong reluctance to share data with certain institutions (such as employers, health insurance) due to fear of genetic discrimination. In Korea, however, Kim et al. found that public interest toward establishing a citizen participation cohort is very high.

In conclusion, we observe that although general access to personal genome data is becoming more widespread, the benefits of such advances are being deployed unevenly. Tools are being implemented that help facilitate the interpretation of personal genomic data and their increased, more secure sharing. We see these advances being undertaken both by academic and industry sectors. But a number of ethical challenges persist, including how to return data to participants in different regions of the world, or how to access direct-to-consumer services and raw data for personal genome analysis, which still remain biased depending on the individual's local jurisdiction. It is our wish to raise awareness about these hurdles and to bring all stakeholders involved into fruitful discussions to promote greater access to the benefits of personal genomics for all.

## Author Contributions

MC wrote the paper with contributions from all authors.

## Conflict of Interest

At the time of writing, MC is associated with Cambridge Precision Medicine Limited. The remaining authors declare that the research was conducted in the absence of any commercial or financial relationships that could be construed as a potential conflict of interest.
